# Expulsion of a Uterine Polypus

**Published:** 1858-04

**Authors:** 


### EXPULSION OF A UTERINE POLYPUS, UNDER THE CONJOINT INFLUENCE OF ERGOT AND BELLADONNA,

BY DR. BEZENCENET.

After the author had given ergot to expel a polypus which appeared at the os uteri, with no other effect than pain and a closure of the mouth of the womb on its contents, he gave an injection per vaginum of the infusion of belladonna, so as to bathe the os in that fluid pretty thoroughly. After this, with the continued use of the secale, he had the satisfaction of seeing on the second day the polypus completely expelled from the uterine cavity. Dr. Beck has also seen the same result follow from these means in one case.—*Ibid.*

